# Rebuilding community resilience in a post-war context: developing insight and recommendations - a qualitative study in Northern Sri Lanka

**DOI:** 10.1186/1752-4458-7-3

**Published:** 2013-01-11

**Authors:** Daya Somasundaram, Sambasivamoorthy Sivayokan

**Affiliations:** 1 Department of Psychiatry, University of Jaffna, Jaffna, Sri Lanka; 2 University of Adelaide, Adelaide, Australia; 3 Department of Health, Jaffna, Sri Lanka

**Keywords:** Collective trauma, Community resilience, Post-war, Post-conflict, Psychosocial, Community based interventions

## Abstract

**Background:**

Individuals, families and communities in Northern Sri Lanka have undergone three decades of war trauma, multiple displacements, and loss of family, kin, friends, homes, employment and other valued resources. The objective of the study was understanding common psychosocial problems faced by families and communities, and the associated risk and protective factors, so that practical and effective community based interventions can be recommended to rebuild strengths, adaptation, coping strategies and resilience.

**Methods:**

This qualitative, ecological study is a psychosocial ethnography in post-war Northern Sri Lanka obtained through participant observation; case studies; key- informant interviews; and focus groups discussions with mental health and psychosocial community workers as well as literature survey of media and organizational reports. Qualitative analysis of the data used ethnography, case studies, phenomenology, grounded theory, hermeneutics and symbolic interactionism techniques. Quantitative data on suicide was collected for Jaffna and Killinochchi districts.

**Results:**

Complex mental health and psychosocial problems at the individual, family and community levels in a post-war context were found to impair recovery. These included unresolved grief; individual and collective trauma; insecurity, self-harm and suicides; poverty and unemployment; teenage and unwanted pregnancies; alcoholism; child abuse and neglect; gender based violence and vulnerability including domestic violence, widows and female headed-household, family conflict and separation; physical injuries and handicap; problems specific for children and elderly; abuse and/or neglect of elderly and disabled; anti-social and socially irresponsible behaviour; distrust, hopelessness, and powerlessness. Protective factors included families; female leadership and engagement; cultural and traditional beliefs, practices and rituals; and creative potential in narratives, drama and other arts. Risk factors that were impeding community rehabilitation and recovery included continuing military governance, depletion of social capital particularly lack of trust, hope and socio-economic opportunity structures for development that would engender a sense of collective efficacy.

**Conclusions:**

In view of the widespread trauma at the individual, family and collective levels, community based programmes to increase local awareness, knowledge and skills to deal with common mental health and psychosocial issues; and training of community level workers and others in basic mental health and psychosocial problem solving are recommended to rebuild family and community agency and resilience. The use of cultural practices and school based programmes would rekindle community processes.

## Background

Disasters, whether natural, for example after a tsunami, or manmade as in war, are now recognized to cause a variety of psychological and psychiatric sequelae [1]. These could range from adaptive and resilient coping responses in the face of catastrophic events to understandable non-pathological distress as well as a number of maladaptive behavioural patterns to diagnosable psychiatric disorders. Conditions like Acute Stress Reaction (ASR, the old disaster syndrome), Posttraumatic Stress Disorder (PTSD), depression, anxiety, somatoform disorders, alcohol and drug abuse have been shown to occur after disasters [1,2]. Chronic long-term trauma can lead to complex PTSD [3], enduring personality changes [4] or Disorders of Extreme Stress Not Otherwise Specified (DESNOS) [5].

However, there is less recognition or understanding of the effects disasters have at the supra-individual family and community levels [6-8]. Less is known about appropriate interventions at collective levels [9]. There are many reasons for this relative deficiency. First, the field of disaster studies is itself rather young. For example, the diagnosis of PTSD was accepted only in 1980 with the American DSM III [10] following extensive experiences with Vietnam war veterans.

Secondly, modern psychology and psychiatry as it has developed has had a western medical illness model perspective that is primarily individualistic in orientation [11]. Geertz describes the Western concept of the individual self as “…*a bounded, unique, more or less integrated motivational and cognitive universe, a dynamic centre of awareness, emotion, judgment, and action organized into a distinctive whole and set contrastively both against other such wholes and against its social and cultural background....is a peculiar idea within the context of world cultures*” [12]. PTSD is constructed as a condition that exclusively afflicts the individual self, the traumatic event impacting on the individual psyche to produce the PTSD. The *World Health Report 2001,* while pointing out that there is considerable mental morbidity among those exposed to severe trauma, warns that there is controversy regarding the cross-cultural validity of PTSD [13]. It has been argued that PTSD is a recent western construct that does not apply in non-western societies [14]. It is being increasingly recognized generally that we need to go beyond the individual to the family, group, village, community and social levels if we are to more fully understand what is going on in the individual, whether it be his/her development, behaviour, perceptions, consciousness, experiences or responses to stress and trauma as well as design effective interventions to help in the recovery and rehabilitation of not only the affected individuals but also their families and community [15-18]. In collectivistic societies, family or community members may join together in *collective coping* to pool resources, act cooperatively sharing the burden to resolve a single or common problem at the family (extended family) or community levels.

For when the family and/or community regained their equilibrium and healthy functioning, there is often improvement in the individual member’s wellbeing as well. Family and social support, networks, relationships and the sense of community appear to be a vital protective factor for the individual and their families and important in their recovery. Cultural rituals and practices, like the North American Sweat Lodge ceremony [19], *thuku kavadi* in Northern Lanka [20] and Eastern Lankan oracle tradition [21], can heal and provide meaning to suffering after trauma. It is also becoming clear that social and cultural values, beliefs and perceptions will shape how traumatic events impact on the individual, family and community and the way they respond [22,23] . The meaning attributed to the event(s), the historical and social context, as well as community coping strategies determines the impact and consequences of trauma. Similarly, firm traditional and religious beliefs and social support has been shown to be a protective factor against the effects of trauma. Equally, community coping and resilience help individuals and families deal with and recover from the destructive effects of disasters.

Unfortunately, there are no clear evidenced based interventions applicable to post disaster situations. Cognitive Behaviour Therapy (CBT) and pharmacotherapy for post trauma consequences like PTSD, Depression and Anxiety are now used in western countries [24-26], but other experts have been critical [27]. On the other hand, once popular and routine methods like debriefing have been shown not to be helpful, even harmful [28]. The Western tradition of seeking help from a counsellor or psychologist would be culturally inappropriate in a collectivistic community [29]. Equally, Cognitive Behaviour Therapy (CBT), the most validated psychotherapy for PTSD in the western world, may not be applicable in non-western communities [30]. Particularly in a low income and poor resource settings with lack of trained mental health workers and with massive populations that have experienced trauma, western individual therapies would not be feasible, while public mental health, community based and culturally sensitive methods would be more appropriate [31]. The widespread problem of collective traumatization or ‘*loss of communality’*[32] following disasters is best approached through community level interventions, particularly in collectivistic, sociocentric communities [33,34]. Further, community based approaches will enable one to reach a larger target population as well as undertake preventive and promotional public mental health activities at the same time. Individuals and families can be expected to recover and cope when communities become functional, activating healing mechanisms within itself. There have been more recent consensus for best practice in community level interventions for massive trauma [7,9,35]. The WHO [36] and other international organizations have come up with the Sphere Project Humanitarian charter and minimum standards when dealing with mental and social aspects of health [37]. A worldwide panel of trauma experts [28] have identified restoring connectedness, social support and a sense of collective efficacy as essential principles in interventions after mass trauma.

The Inter-Agency Standing Committee (IASC) Guidelines on Mental Health and Psychosocial Support in Emergency Settings developed by the United Nations with the experiences and collaboration of over 200 organizations working worldwide in situations of mass trauma [38] recommend considering the socio-political and cultural context to maximize the participation of local populations, building on available local resources and capacities and integrating close collaboration between support systems when responding. The construct of social capital is becoming increasingly recognized as an important factor in mental health [39,40]. Social capital is protective for mental health and wellbeing. Disasters such as a chronic civil war can lead to depletion of social capital [41,42]. Bracken and Petty [43] argue that in current interstate conflicts, “*Civilians are no longer ‘incidental’ casualties but the direct targets of violence*. *Mass terror becomes a deliberate strategy. Destruction of schools, houses, religious buildings, fields and crops as well as torture, rape and interment, become commonplace. Modern warfare is concerned not only to destroy life, but also ways of life. It targets social and cultural institutions and deliberately aims to undermine the means whereby people endure and recover from the suffering of war…”.* In trying to design post war recovery programmes, it becomes important to try and understand the dynamics of how social capital is destroyed as well as look for ways to rebuild it. According to Colleta and Cullen [44],

“*Unlike interstate conflict, which often mobilizes national unity and strengthens societal cohesiveness, violent conflict within a state weakens its social fabric. It divides the population by undermining interpersonal and communal trust, destroying the norms and values that underlie cooperation and collective action for the common good, and increasing the likelihood of communal strife. This damage to a nation's social capital, ­ the norms, values, and social relations that bond communities together, as well as the bridges between communal groups (civil society) and the state ­ impedes the ability of either communal groups or the state to recover after hostilities cease. Even if other forms of capital are replenished, economic and social development will be hindered unless social capital stocks are restored*”… Such an understanding could enhance the abilities of international actors and local policymakers to more effectively carry out peace building-relief, reconstruction, reconciliation, and development in the post-war context.

**Figure 1:**
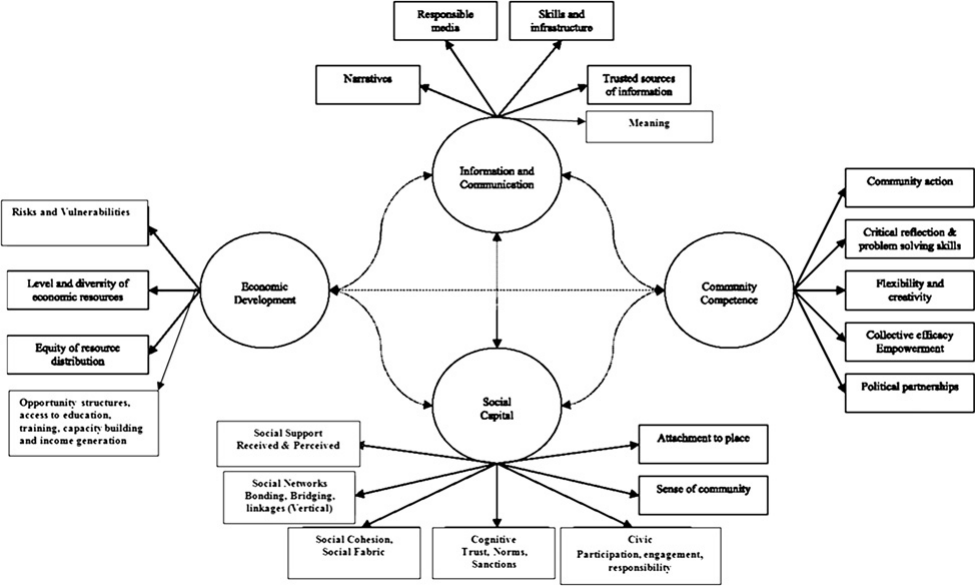
**Community Resilience as dynamic, networked adaptive capacities **[108]**.**

The strategies for reconstruction and revitalization of social capital after conflict that have been recommended include strengthening social networks and community ties, building social organizations, and macro-social policy reform to increase community access to external resources and power [45]. At the same time as repairing the destroyed social capital, it is vital to preserve, foster and promote the natural resilience of communities to recover and help themselves. Community resilience can be seen as positive, collective adaptability despite high levels of adversity; in this case, the impact of the natural disasters, such as the Asian Tsunami, and the manmade disaster, such as the chronic civil war. An excellent conceptual framework (Figure 1) for building community resilience as a process of dynamic adaptation with a positive trajectory to buffer the adverse effects of disasters and promote community wellbeing has been developed [46]. It is suggested that it is important to ensure commitment, engaging the entire system of the community in an inclusive process; identify scripts, themes and patterns across generations and community history; foster creativity as the central process of healing maintaining sensitivity to issues of culture, gender and spirituality; and encouraging access to all natural and ancillary resources. Other factors mentioned include building on existing resources; collaborating and networking across all systems; relating programme needs to goals, future and best interests of the community; encouraging natural change agents and leadership within the community; empowering families and communities; and developing ownership by the community [47].

The analysis of social protective and risk factors and the social dynamics of coping and growth can help illuminate social processes and identify collective competency factors that can be built upon. Important characteristics of resilient communities include availability of family, kin and neighbourhood support systems and networks (i.e. bonding social capital), community resources like respected and functioning elders, traditional healers and cultural practices, religious leaders and organizations, institutions like schools, health facilities, governmental and non-governmental organizations, community level conflict resolving mechanisms and functioning structures like the judicial system, democratic practices and access, free media, and reliable information. Economic and income stability, employment, occupations and traditional vocations, food, shelter, security, and other essential needs being met would help communities cope with adversities and shocks to the system. Norris et al. [46] identify four primary sets of adaptive capacities for community resilience — economic development, social capital, information and communication, and community competence (Figure 1). Community competence refers to the capacity, resources and skills within the community to act together, cooperatively and effectively, to meet challenges. Unfortunately in disaster situations, particularly chronic war contexts, some or many of these resources and support systems would be affected, dysfunctional or not available. Community responses and coping may thus become compromised. A vicious resource loss cycle [48] where breakdown of social support, networks, leadership, economic resources and supply chains of material goods, will create a downward spiral of a deteriorating situation of increasing needs and dysfunction.

Community values, beliefs and traditions can provide bulwarks against mass trauma [15]. Adversity-Activated Development (AAD) describes salutary social transformation, post-traumatic growth and progressive changes following challenging circumstances [49]. Thus the breakdown of traditional forms of oppression and rigid hierarchical structures like caste, feudal ownership and patriarchal suppression of women could lead to more positive emancipation and development. New organizations, networks, relationships, friendships, forgetting of old quarrels and conflicts, shared memories and experiences could lead to community growth and enhancement. Motivated and vibrant leadership may emerge while older, ineffective and anachronistic methods are shed. There can be radical and revolutionary alteration in the social trajectories due to critical challenges. Common challenges can forge social unity and cohesion [50]. The common people, and oppressed and excluded minorities, could gain more power and access to resources due to shifts in the social system or out of collective action. Collective consciousness can be awakened leading to more awareness and knowledge. In the tradition of Friere’s ‘conscientisation’ [51], the breakdown in social structures and institutions creates an opportunity for empowerment, collective transformation and re-alignment of social dynamics, “*challenging existing structures of power and achieving a shift in power relations, ultimately resulting in the transformation of the existing social order*” [52].

**Figure 2:**
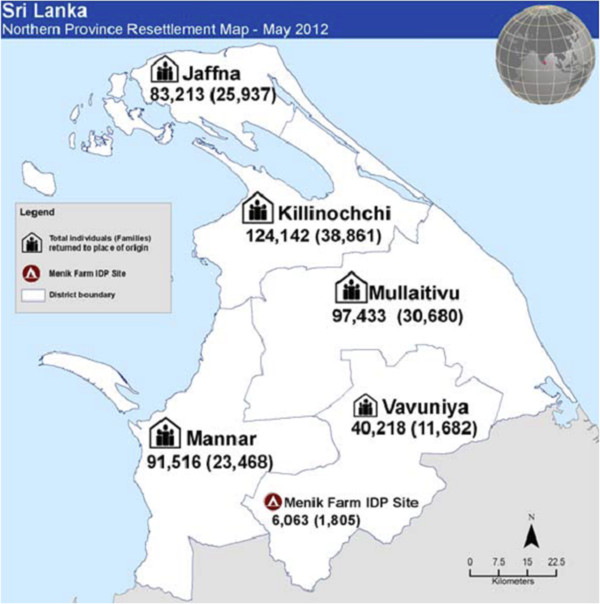
**Resettlement of IDP’s in the Northern Sri Lanka **[75]**.**

The civil war in Sri Lanka typified in many ways the global trend towards intrastate conflict [53,54] where ethnic passions [53,55-61] and inequalities play a pivotal role [62]. The bitterly fought contest created a casualty list of well over 100,000 killed; and many more injured, mentally affected, displaced both internally and overseas; and communities, property and ecosystems destroyed. The Sri Lankan state, the various Tamil militants, in particular the Liberation Tigers of Tamil Eelam (LTTE), which for over two decades fought to create an separate state, the Sinhala Janatha Vimukthi Perumana (JVP), an ultra-leftist militant group that made two attempts violently to overthrow the government, and India, during its short intervention in the island (1987–90) to impose peace, were all involved in a dirty war [63,64] with grave human rights violations and crimes against humanity [65,66]. Though critical at times, the international community, its many organizations, diplomatic missions, UN, and aid agencies giving technical support, military hardware, training and the global network of socioeconomic ties and mutual relationships that give covert recognition, legitimacy and tacit sanction as well as the Sri Lankan and Tamil diaspora communities that supported the conflict were also indirectly implicated [63,64]. Although the actual physical fighting ended dramatically in the Vanni in May, 2009, with the state using indiscriminate shelling and bombing as the LTTE held the civilian population hostage that resulted in over 40,000 civilians deaths, many more injuries and unacknowledged war crimes [67-73]; the underlying ethnocentrism and political causes remain unresolved. The survivors of the Vanni massacre were herded into barbwire internment camps with poor facilities and tight security. With increasing pressure from the UN and IC, the state has gradually relaxed its grip, but the north and east remain virtually under martial law [74]. Very few external, but mostly internally displaced populations (IDP’s) have been resettling in their original areas from 2010. By 30 June, 2012 about 440,700 people (131,000 families) had returned to the Northern Province. This figure includes some 230,000 people (72,000 families) displaced after April 2008, that is during the recent Vanni war; and 211,000 persons (58,000 families) displaced before April 2008, that is the more longer-term IDP’s including Muslims and Sinhalese (see Figure 2) [75]. However, some have not been allowed to return to their original homes and lands due to continuing militarized High Security Zones (HSZ). There is some economic and infrastructure development in the war torn areas [76], but three years after the ending of the war, legitimate reparation, community recovery and national reconciliation are yet to take place. The community and its members need to be able to benefit from the developmental programmes being undertaken. Economic recovery will not be sufficient, people need ‘to reconstruct communities, re-establishing social norms and values’ [77]. International law recognizes the Principle of Restitutio ad integrum for the redress of victims of armed conflict to help them reconstitute their destroyed ‘life plan’ [78,79]. This justifies the need for rehabilitation as a form of reparation clarified by the UN ‘Basic Principles and Guidelines on the Right to a Remedy and Reparations for Victims’ as taking five forms: restitution, compensation, rehabilitation, satisfaction and guarantees of non-repetition [80]. This should necessarily include psychosocial rehabilitation [81].

Individuals, families and communities in Sri Lanka, particularly in the North, the East and so called border areas of Sri Lanka, have undergone twenty five years of war trauma, multiple displacements, injury, detentions, torture, and loss of family, kin, friends, homes, employment and other valued resources [7]. In addition to widespread individual mental health consequences [82,83], such as PTSD (13%), anxiety (49%) and depression (42%) in the recent Vanni IDP’s [84]; families and communities have been uprooted from familiar and traditional ecological contexts such as ways of life, villages, relationships, connectedness, social capital, structures and institutions [85]. The results are termed collective trauma which has resulted in tearing of the social fabric, lack of social cohesion, disconnection, mistrust, hopelessness, dependency, lack of motivation, powerlessness and despondency. The social disorganization led to unpredictability, low efficacy, low social control of anti-social behavior patterns and high emigration which in turn causes breakdown of social norms, anomie, learned helplessness, thwarted aspirations, low self-esteem, and insecurity. Social pathologies like substance abuse, violence, gender based- and child- abuse have increased.

The study attempts to understand the mental health and psychosocial problems faced by families and communities in the current realities of a post war context in northern Sri Lanka, analyze the risk and protective factors to recommend ways of addressing these needs based on expert consensus on best practice, our previous experiences gained during the war [86,87] and after the tsunami [85,88], as well as findings from the study.

## Methods

This is a qualitative, ecological study using critical psychosocial ethnography. The study uses as its framework the ecological model of Bronfenbrenner [89] with the micro, meso, exo and macro systems or the individual nested in the family nested in the community [48,90], focusing more at the family and community levels. The WHO definition of health [50] can be adapted to the Bronfenbrenner model which also emphasizes the need to look beyond the micro or individual and physical level:

"*Health is a state of complete physical, mental, (familial), social, (cultural), (spiritual) and (ecological) well-being, and not merely an absence of disease or infirmity".*

- World Health Organization (WHO)

The family unit (in parenthesis for author additions) has been included as it is paramount in traditional Tamil society while the spiritual dimension is an essential part of the Tamil culture. The spiritual dimension has been put forward at various WHO fora but has not been formally accepted yet. Culture is increasingly recognized as an important dimension of mental health [91]. The ecological dimension arises from Bronfenbrenner’s and environmental models and systems theory that emphasis an overall holistic approach, looking at how the different levels, dimensions and systems with different temporal trajectories of their own; influence each other to produce an interactive, dynamic (dys)functional whole. The disaster itself has an impact on these systems and their interaction, and moreover has a temporal trajectory of its own [92,93]. More recently a growing consensus has been emerging on the need to look at these wider dimensions to understand the dynamics of the effects of disasters and to design effective interventions [9,18,94]. This study took place in the long-term post disaster phase, usually lasting years, when traditionally recovery, resettlement, reparation, restoration, rebuilding, rehabilitation, and development are expected, promoted and implemented.

This critical, psychosocial ethnography seeks to describe and understand the lived experience of ordinary people in a post war context in northern Sri Lanka from their inner psychology, outer behaviour, social structures and dynamics using psychological and sociological introspection [95] and phenomenology. It critically inquires [96] into the pressing psychosocial issues people face in their daily lives and the inequities in power and access to resources, in an attempt to find ways to address some of these problems, an adaptation of the public ethnographic method [97]. It is also an autoethnography, where the authors are ethnic Tamils, natives or insiders critically observing their own community for subjective and intuitive insights into cultural, religious and social contexts, events, attitudes, values and behavior of their communities [98,99]. The cases for in-depth study were selected to represent common experiences of the community in general, eschewing the extremes and more sensational accounts, so that a collective narrative emerges. Thus the ‘epistemological and ethical authority of testimonial narratives’ gives rise to their authentic metonymic property to reflect the whole community [100]. In-depth interviews of individual cases and their families, supplemented by observations of their environment and interactions by home visits and interviews with significant others, produced detailed description and helped understanding the complexities of lived lives, problems faced and manifested, how they coped and survived and the context. In cases of suicide, the technique of verbal autopsy [101] where intimates of the deceased are interviewed and relevant medical including the medico legal post-mortem reports by the Judicial Medical Officers (or his deputy) ordered by the Coroner, and other documents were examined, was employed.

The setting was Northern Sri Lanka in the first half of 2012, comprising Jaffna, Killinochchi, Mullaithivu and Vavuniya Districts. Data was obtained through participant observation; case studies and Key Informant (KI) interviews; and Focus Groups (FG) discussions. Participant observation of the general population of this area, their day to day life, psychosocial functioning, context and dynamics was done while working in the mental health field and compared to earlier periods, before and during the war as well after the tsunami [7,8,102,103]. The overall methods adopted here were very similar to Brandon Hamber’s [104] research into the post-Apartheid South African experience:

“*Thus, I cannot escape the fact that the research …. is built on active participation in the area under study. I was an observer, a participant, a researcher, an activist, and an action researcher. My position in relation to the area under study and the research that flowed from it cannot be divorced from my role in the process. Furthermore, the research produced and presented here was undertaken in a dynamic context of political and social upheaval in the country at the time. Valuable evidence of how the social world operates can also be generated by observation and participation in interactive situations and social settings. Broadly I adopt a perspective that might be described as “emic”; that is understanding phenomena on their own terms, with insights flowing from inductive processes, and building general accounts from pieces of experience and research. Actors immersed in the context know what the experience of that social setting feels like… and in that sense they are epistemologically privileged*”.

Case studies and in depth clinical interviews were carried out by the principal author and by trained mental health workers of subjects during their normal psychosocial work. Sampling of respondents and informants was purposive and convenient, where they were available such as in mental health clinics or during field work and deemed interesting. Some were referred by community and village resources, while some were selected by using the snowball technique of following up those identified by cases as having similar psychosocial problems. Cases, KI’s or FG members were not compensated in any way. Key informants included mental health and psychosocial workers, government and non-governmental (NGO) officials, teachers, priests and community leaders. Sixty cases were studied in depth, 35 KI’s were conducted during this period. Seven FG discussions were carried out with mental health and psychosocial community workers separately in Jaffna, Killinochchi, Vavuniya and Mullaithivu and two with teachers and one with women development workers was undertaken in Jaffna. On the average there were 20 participants at each FG and ran from two to three hours. A workshop on psychosocial problems in students with 40 teachers from all over the north lasted a whole day. Data collection was done under risky conditions. Psychosocial work or research were not officially permitted and had to be done with circumspection under some other guise without raising suspicion. Any kind of recording and writing could come under scrutiny. As such data collection was done discretely and manually, sometimes with jotted cryptic notes or committed to memory for later transcription. The case studies were structured assessments involving a mental state examination and ecological observations of context, relationships and social environment. The KI interviews used semi-structured, open-ended probes. Participation observations was ethnographic and ecological. Triangulation (using more than one qualitative method including documental analysis, media and organizational reports) was used for multiple perspectives, to check conclusions and broaden the analysis to reduce bias. Participatory Action Research (PAR) methodology was used to feedback transcripts, documentations, findings, conclusions and recommendations to participants for corrections, comments and suggestions, sometimes by e-mail or telephone, which were then used to modify and change the findings and discussion as an ongoing, interactive living process. The FG discussions were in Tamil. Most KI interviews of local participants were also in Tamil but a few with International NGO members were in English. Translations from Tamil were done by the principal author. Suicide and attempted suicide data up to 2012 was obtained through hospital sources, courts and Auditor General Department reports.

Qualitative techniques used eclectically to analyze the data by the authors included case studies, phenomenonology, grounded theory, symbolic interactionism and hermeneutics [105]. Data analysis was done manually. The case studies or the collective case study [106], KI’s and transcripts of the FGD were used to elicit categorical aggregation, find common themes and similarities, direct interpretation and generalizations. The phenomenological analysis sought to understand the meaning, structure and essence of the lived experience for a community while grounded theory is used to look at psychosocial structural processes to develop propositions, conditional matrix, alternate interpretations, themes, hypothesis, and theory. The trauma grid [49] was adapted as a tool to organize the data and conceptualize the functioning of the community in the context of different levels: individual, family, community and society/culture, as these may be affected in different ways, positively, negatively or varying combination of both. Techniques from hermeneutics helped to interpret the political, historical and sociocultural meanings while symbolic interactionism considered the symbols, metaphors and idioms of distress for the multiple meanings of psychosocial interactions. The overall attempt was to extract the meaning and implications, establish themes and patterns to reveal a composite picture of the lived situation, context and shared psychosocial problems so as to find ways to help with effective interventions [107].

Ethical clearance for the ongoing study was sought from the Ethical Review Committee of the University of Jaffna. As open research or assessment of needs in this population were not being permitted, the participants, assistants, sources of information and organizations have been left anonymous.

## Results & discussion

The common psychosocial problems and adaptive changes that were observed and brought out by the KI and FG discussions were inserted into a Trauma Grid [49] adapted for the Northern Sri Lanka context (see Table 1). The findings were divided into expected ordinary reactions to human suffering, more distressful psychological suffering which would benefit from community and family support and diagnosable psychiatric disorders needing professional help. These could be understood as a continuum and not discrete categories with rigid boundaries of exclusion. Positive effects included resilience and beneficial transformative or adaptive change. The identified changes were located as belonging to the individual, family, community or social/cultural levels. Some responses overlapped, saddling several levels.

**Table 1:** **Trauma Grid** (**adapted for Sri Lanka context from**[19]

	**Negative effects**	**Positive effects**			
**Levels**	***Ordinary human suffering***	***Distressful psychosocial reactions***	***Psychiatric disorders***	***Resilience***	***Adversity Activated Development***
***Individual***	Sorrow, worries, normal grief, fear, stress, anger, uncertainty, magical thinking, psychological trauma, injuries, handicap, losses, low educational attainment	ASR, intense and extreme levels of suffering, complicated grieving, adjustment disorders, maladaptive coping, alcohol & drug (including non-prescription medication) use, somatization, help seeking behaviour, change in ideology/faith, fear of future, suicidal thoughts/behaviour	PTSD, Depression, Anxiety disorders, Prolonged Grief Disorder, Alcohol & Drug Abuse, Complex PTSD, DSH, Brief (reactive) psychosis, Dissociative episodes, Personality disorders	Independent, mature personality, adaptive coping mechanisms, flexibility, establishing and maintaining relationships, planning for their future. socialization and networking skills, entrepreneurship	Post Traumatic Growth, female leadership, empowerment, liberation, creative activities, nontraditional thinking, innovativeness, nontraditional jobs
***Family***	Displacements, separations, deaths, handicap, loss of properties and structures (buildings), disappearances, orphans, single parents, family disharmony, break-up of extended family system	ASR, grief, family conflicts, domestic violence, separations, divorces, extra-marital relationships, unwanted pregnancies, child & elder abuse, poor parenting, scapegoatism	Dysfunctional family units, morbid jealousy, family pathology, child psychiatric disorders or emotional and behavioural problems among children, homicide- suicide pack	Unity of nuclear families, cohesion, extended family ties, support system, new relationships, goals and aspirations	Functional female headed households, diversity in marriages, Split families
***Community***	Displacements, up rootedness, separations, destruction of normal systems and structures, dysfunctional structures & institutions, loss of buffer system, reshuffled neighborhood, depleted social capital, poverty and unemployment/ underemployment	ASR, denial, rationalization, intellectual dissonance, hopelessness, helplessness, powerlessness, herd instinct, silence, suspicions, distrust, uncertainty, breakdown of ethical and moral values, catharsis, sexual abuse	Collective trauma, suicide, mass hysteria, impulsiveness and antisocial behaviours.	Rituals, revival of traditional arts (koothu), ceremonies, remembrance observations, monuments and grave stones, social functions	Acceptance of female leadership, female empowerment & liberation, new ways of thinking and breaking of traditional boundaries, entrepreneurship, awareness of global trends, emerging new form of arts (like cinema, short films), meaningful narratives, practical (problem solving) support, micro finance schemes and economic development
***Society****/****Culture***	Depleted social capital, dysfunctional structures & institutions, patronage, authoritarian personalities, corruption	Hopelessness, helplessness, powerlessness, silence, suspicions, distrust	Collective trauma, suicide	Rituals, ceremonies, remembrance observations, social functions, increasing tolerance about others view, culture and life style	Reduction of caste barriers, female leadership, empowerment, liberation, multi-cultural milieu, rights oriented thinking and behaviour

There was widespread exposure to potentially traumatizing events that under normal conditions would be considered extreme and would cause distress in most people. Commonly these traumatic events had been multiple and chronic. Thus forced displacement in extreme situations was universal and commonly multiple (up to 10 or more in many cases). The unexpected and sudden death of a close family member(s), relation(s), and friend(s) in distressing ways was again almost universal experiences. Experiencing injuries, disappearances, separations, internment, arrests, detentions, beatings, bombings, shellings, shootings as well as witnessing these events were common. Undergoing extraordinary physical hardships like thirst, hunger, long marches, and lack of medical attention or shelter were experienced by most families and communities. As such these experiences were considered for the purpose of this study as norms for the population and placed under ‘Ordinary human suffering’. When these experiences caused observable behaviour or complaints of a psychosocial nature amounting to distress they were placed under ‘Distressing psychosocial reactions’. When the signs and symptoms met criteria for a diagnosable condition, they were categorized as ‘Psychiatric disorders’. Where individuals, families or communities showed any positive response, coping, adaptation or growth, these were placed under ‘Resilience’ or ‘Adversity Activated Development (ADD)’. Under the circumstances, the lack of adverse reactions to these extraordinary experiences, termed here as ‘Ordinary human suffering’ could be considered positive coping. These categorizations have pubic mental health implications and how it is dealt with, particularly in a resource poor setting. Several participants were appreciative of this conceptual model when it was fed back and commented that it would be a useful tool for organizing and planning their work in the field.

It was initially difficult to bring out positive aspects in the KI interviews and FG discussions. Only with persistent probing and directive questioning were adaptive changes and resilient factors mentioned. There may have been an expectation that negative aspects were being looked for. There may have been a lack of appreciation of positive developments. Most of the PS and health workers had been trained to find pathology, signs of disease, not wellbeing. It was going against the grain of how they usually perceived and operated. But it may have also been difficult to discover anything positive, hopeful in the situation. It could have rather been that by identifying the problems, the hindrances to resilience and recovery could be addressed, the deficiencies, the blocks removed and thereby resilience rebuilt. The open ended FG and KI discussions tended to elicit common PS problems they were encountering in the field. On further questioning they mentioned positive adaptive changes they were seeing. Through the process of the study, directive questioning and feedback discussions, it appeared that participants gained an increasing awareness and appreciation of the positive changes and saw the possibilities of promoting these adaptive changes in their work.

The findings from the participation observations by the authors, KI interviews and FG discussions are reported, analyzed and discussed under the headings Individual, Family and Community, as well as significant special topics under Suicide, Grief and Governance.

### Individual

At the individual level, there were persons with PTSD, Depression, Anxiety related conditions, Alcohol abuse in males, Somatoform disorders, suicide and attempted suicide. Most of them did not seek psychiatric or mental health help but were found to request help during community visits. The majority appeared to have accepted these responses to their experiences in the war as normal. However, they had signs of disability, reduced motivation, sleep problems, nightmares, somatic complaints, fear, insecurity, easy startling, and other symptoms. Without adequate outlet or healing, it is possible that the trauma was manifesting itself as somatic complaints and other idioms of distress [97] as well as causing problems and conflict in their families and communities. It is possible that with appropriate psychosocial interventions, opportunities to share and receive support as well as use their emotions in more constructive and creative ways, most of these negative reactions could be turned around towards a more positive trajectory.

At the same time, there were exceptional individuals, particularly youths, showing independence, mature personalities, adaptive coping mechanisms, flexibility, establishing and maintaining relationships, particularly across other groups, socialization and networking skills; planning for their future and entrepreneurship. Some had shown remarkable courage during the war, rescuing, caring and helping others. They had survived and coped through astonishing danger and destruction. Leadership, innovation and management skills had developed in some, particularly females and youth. There was evidence of post traumatic growth in their personalities and functioning. However, these youth showing motivation and developing skills in dealing with world, often lacked opportunities structures in a regularized and systemic way for meaningful income generation activities, vocational training, employment or further education. It could be observed that many were becoming frustrated and at a loss of how to advance or even contribute to their family and community welfare. It was evident that the communities and their leaders lacked access to the resources and power structures to harness this available potential for the benefit of the community. Some participants and KI’s felt that the observed discontent among youth, increasing antisocial behavior and problems, including those within families and communities, were a manifestation of the blocks to advancement, a feature of the post war context, rather than the effects of war trauma per se. Some elderly KI’s warned that the long term political fallout could prove grave. At the same time, providing for appropriate means of advancement and development would also help in the healing and recovery process.

### Family

At the family level, conflicts, separations, remarriages, extramarital arrangements, morbid jealousies and abandoned children were leading to break up of families and family dysfunction: many participants reported noticeable increase in teenage and unwanted pregnancies; alcoholism among males; gender based violence and vulnerability including domestic violence leading to a host of secondary psychosocial problems. There were many widows and female-headed households struggling to make ends meet and keep their families going. The burden of responsibilities had fallen on the shoulders of these women who showed an increased incidence of MHPS problems as a consequence. The loss of a family member, particularly the male, earning head of the family was a risk factor for MHPS problems. Some families were dealing with chronic, often inoperable physical injuries and handicap in a member. Children and elderly were vulnerable groups with special problems, particularly within dysfunctional family settings. There was a noticeable increase in reports of child abuse and neglect; poor parenting, family pathology, and scapegoatism, that is child psychiatric disorders or emotional and behavioural problems where the child had become the conduit through which the abnormalities in the family dynamics was manifesting.

Some families were being driven to homicide-suicide pacts. Parents forced their children to drink insecticide before they took it themselves.

Couples too committed suicide together. A case in Mullaithivu revealed how past war trauma, grief, and current resettlement stress intertwined with social issues such as caste :

Kavitha was born in 1988 at Kumulumunai. Her father was killed in 1991 during a Kfir bombing. She lost her mother in 2009 during the final battle at Mullivaikal. Being the only child, her maternal uncle took responsibility for her. Rajah was born in 1983 at Murippu to a family with four siblings. While studying A levels, he was compelled to join the LTTE. In 2009 he lost a leg and tried to commit suicide by biting his cyanide capsule but survived. He was detained in 2009 and released in 2010. Kavitha had been depressed in the IDP camp, grieving for her mother. When Rajah was released to the IDP camp, he had met Kavitha there and both fell in love. The stepbrother of Kavitha had disowned her saying Rajah was of low caste. They had then been resettled in Mullaithivu. According to his relations, Rajah was greatly affected by being handicapped. He often spoke about dying saying it was no use living like this. He was very irritable. He would often damage his leg prosthesis out of despair. After Kavitha had been with them for some time, Rajah’s family started getting into frequent arguments with her. On the night of the suicide, both had dressed in new clothes and, with a picture of the God Anjeneya, had gone into a nearby forest. They had eaten ‘vadai’ and drunk together a soft drink mixed with insecticide. They had tied their legs with Kavitha’s Punjabi shawl which was then wrapped tightly around both their waists to hold them embracing each other in death. When Rajah’s mother was asked why she had not contacted the guardian of Kavitha beforehand, she had smiled ruefully saying that they could not/were not allowed to associate with ‘that side’. According to a village leader, Kavitha was of high cast (vellala) while Rajah was of low caste.

On the positive side in the North, a majority of families were still united, particularly at the nuclear level. There was mutual support, with hierarchical and shared responsibilities; they faced challenges together, resettled and rebuilt their homes; sent children to school, derived whatever benefits and aids that were available; and found employment and income with a unity of purpose. There was cohesion in their functioning. Families attempted to return to their own homes and rebuild their destroyed homes with whatever was available or obtain aid and material to do so. They also planted trees, cleaned their land grew livestock and attended to their agricultural work. Community resilience is based on the attachment to place, be it home *(veedu)*[99], village (*uur*) or region (*thesam)*. The resettling and living in their old home or village has a healing effect [108] that can lead to recovery [77]. In the traditional Tamil identity *veedu* and *uur* were central to their self-image, confidence and resilience [109].

Many families still maintained their extended family ties, support systems, and relationships, which were all protective, increasing social support mechanisms . The fundamental cornerstone of Tamil culture, the family structure, had survived the catastrophic events and was functioning strongly. Community resilience depended on the functioning family and interventions for recovery and rehabilitation should be primarily directed at strengthening this basic unit. In fact, some of the female headed families continued to function despite the loss of the key father/husband, breadwinner figure. There had been some diversification in marriage arrangements, allowing for adaptations and flexibility, for example in caste considerations, and widows remarrying or having allegiances of mutual benefit despite the disapproval of traditional cultural mores.

FG discussions brought out one form of adaptation observed in the post-war Vanni resettlement process. An estimated 90,000 widows in the North East, many of whom have lost their husbands to the war, have to rebuild their shattered lives as single headed families. Ostracized by traditional Tamil society and in dire economic straits, they are compelled to depend on the military who control most needs, be it housing, rations, allowances, subsidies or schooling. They are then placed in a vulnerable and helpless position (International Crisis Group, 2011). They are also lonely and in need of help in masculine tasks. Soldiers lend a helping hand in rebuilding their houses in remote villages. Sometimes, they are able to get the land and housing loan, materials and other benefits with their military influence. Natural albeit temporary friendships and liaisons are struck up out of mutual needs and attraction. A child follows and the house and land eventually go to the child but the soldier moves on. The practical arrangement results in mutual benefit. The International Crisis Group (2011) report also mentions some of the returning Muslim men helping socially marginalized widows. There were also reports of prostitution.

Most families had managed to send one or more of its members abroad through innovative strategies and routes who in turn remained faithful, supporting their family economically and helped others to migrate or find their bearings. Some families had split up their living arrangements for work or education purposes, for example one parent and child moving to a city while the rest remained in the village.

### Community

At the community level, our participant observations, collective case study through multiple cases [106], FG discussions and KI interviews revealed the recurring themes of general hopelessness, helplessness, and powerlessness. Though participants appeared relieved by the end of the fighting there were widespread feelings of insecurity in the remote villages of the Killinochchi and Mullaithivu districts (previously controlled by the LTTE, the so called ‘*uncleared areas’*) with the ongoing resettlement process due to the omnipresence of the military. The levels of fear appeared to be less in Jaffna and Vavuniya (which had been under the control of the Sri Lanka army for much longer - the so called ‘*cleared*’ areas). Our observations were that community leaders and members were less articulate, reluctant to participate or voice their opinions compared to earlier times. In general, people appeared to prefer to remain silent and noninvolved. Many active Community Based Organizations (CBO’s) and groups had become inactive or defunct. People were suspicious of each other, distrustful of the intentions of others, no longer willing to put their trust in community organizations. FG discussions and statistics from Child Rights and Gender Based Violence (GBV) organizations revealed increasing reports of child abuse, both physical and sexual, domestic violence, unwanted pregnancies and illegal abortions. It was not clear whether there was a real increase or whether it was due to increased reporting due to better awareness, access to functioning structures like child and GBV desks and active case finding by concerned workers. Alcoholism among males was brought up as a common problem in communities. Alarm was expressed about alcohol and some drug use among youth and students. Thefts, petty crimes, antisocial activities and corruption were more conspicuous. There appeared to be a general perception the ethical and moral climate in communities was deteriorating rapidly compared to how ‘tightly controlled’ (*kaddupaadu)* it had been under the LTTE.

A health specialist complained,

“*Most of the people are self-centered. Educated people are not willing to talk about human rights. It seems to be a natural response to trauma by the university community, and religious, legal and medical leaders. Individually also some people are not worried about what is happening next door even if it is a medical emergency. Sexual abuse and theft are common even in organizations and institutions because there is no one to monitor. Gang behaviour in youngsters has increased. Corruption is common in public works and it’s safeguarded by political influence. On the positive side, some of the people from abroad and Colombo are coming back to settle down here, while some people here want to migrate aboard. Muslims are being resettled in Jaffna and a good relationship is maintained with the local people. But there is blame on Muslims related to drug dealing, abducting and sexual violations. Muslim politicians are threatening the Tamils, especially in the Vanni*”.

He described a surreal atmosphere “*where people deny the actual situation and their past, what had happened in the war, and carry on with their own lives as if nothing was wrong. There is a falsification of memory*.”

FG discussion mentioned examples of mass hysteria, such as aspects of the ‘*grease putham*^a^’ phenomena [110] and attacks of fainting epidemics in schools, that were symptomatic of underlying insecurity. A group of mental health workers who were returning late at night from field work were set upon by a community vigilante groups that had been formed to ward off the alleged *grease putham* attacks and barely escaped being man handled. They were told that was an occupational hazard of working late! Herd instinct in election voting or strikes was mentioned. The exodus of Tamils seeking to escape to Australia by boat was called a mass hysteria [111,112] but a more apt terms would have been herd instinct. It could be observed during this period that ordinary people talked about fleeing to Australia by boat to escape the hopeless situation. A vegetable seller at the Saturday public market told her clients gathered around her that rather than put up with the situation, she was going to flee to Australia where she could make a life for herself and her family.

FG discussions described widespread economic difficulties and poverty due to loss of homes, property and other belongings and that after the war there was inadequate livelihood and sources of income. It was also brought out that families were being resettled in remote villages in the Vanni without adequate resources or material. They were struggling to survive socio-economically. Loss of gainful employment, underemployment, lack of appropriate vocational training for employment and opportunities for commercial enterprise were undermining the recovery process, particularly among youth. FG’s mentioned the vicious cycle of depression where the poverty and lack of opportunities were making once active men and women depressed which in turn made them loose motivation, worry and feel helpless. They were unable to participate or make use of whatever was available. The following two family case histories from Mannar are illustrative of the complex impact of war on current realities:

1. *Mrs. S is a 40 year old widowed mother of six children. Three of them are deceased, and one is missing since the last stages of the war in 2009. She became a widow during the same period, as her husband succumbed to a gunshot injury. Her current family consists of her youngest child, a daughter who is studying for GCE O/L and two grandchildren from her second son who had died along with his wife in the war; the grandchildren are schooling nearby. At present, the only means of income is the occasional odd-job and a minimal income from the home garden. In 2009, during the war, they were gradually displaced towards the north-eastern part of the island. Whilst losing her children and husband, she underwent severe hardship and mental trauma. In fact, her family was a well to do unit before the war, owning land which they cultivated and owned a well-equipped house. She presented to the mental health clinic in 2009 with an episode of severe depression and showed symptoms such as poor sleep, loss of appetite, somatic complaints, poor concentration and interest and suicidal thoughts. She showed marked psychomotor retardation and hopelessness. Her condition is controlled well with the medication. Mrs. S is determined to lift her family up from this hardship. The lack of an effective social support system is very evident. The mental health unit, Mannar is trying to find her financial and social support.*

2. *A family of six were displaced continuously starting in September 2007 from Mannar, then to Killinochchi and finally Mullaithivu Districts. During this displacement in February 2009, the father was injured in the right side of his neck due to a shell and was taken to the Trincomalee hospital by the ICRC ship. During this period the other members of the family were in Vanni. In March 2009, the elder daughter was forcibly recruited by LTTE and still there is no information about her. In April 2009, the mother was killed due to shelling. The rest, comprising three children, were brought into government controlled area in May 2009 by relatives. Now the father and these three children are living in Mannar Adampan area. The children are studying. The father was a blacksmith but he is now unable to work in an efficient manner because of his injury in the right neck. They are struggling to manage their daily life*.

Another history from Killinochchi also brings out the difficulties faced by many families:

“What I am telling you in words is something I, my family, and many others experience atom by atom (anu anuvaha) every day. What I am going to say will bring back past memories. For that do not cry and show your sympathy. We were living happily as a farming family of seven. We owned our own tractor and were doing well. We had the respect of the village. Then tragedy started with the war- (arrakan vanthu puhunthathu). In April 2007, the movement took my son. He was returned in May as a corpse. I died for a moment. I lost my happiness. My heart started to weaken. Afterwards, due to the intense shelling, we started to move from place to place. I became anxious trying to safeguard my remaining four children. My husband also started falling ill but there were no medical help. In April, 2009 he became serious and they took him by the ICRC boat. In a short period we went over to the Army side. They sent us to the camp. We were short of food and water. We didn’t have any money to buy anything for the children. Then I started to search for my husband. I lived with the hope he would be alive. Finally, they confirmed that he had died. My mind became disturbed. I did not know why I should go on living. Then I thought of my children and lived for them. In March, 2010 we were resettled. When we came to our land, I went into shock. There was nothing there; everything had been leveled to the ground. I wailed loudly. I couldn’t take a step into our land. We struggled to get back to normal life. All the children are still studying. We had no one to go for loans. My son left his studies to work in a garage. We ate from what he earned. I continued to live to bring up the rest of my children. I have been taking treatment at the Killinochchi mental health unit for two years but my burden has not gone from my heart.

A KI, a mental health professional working in the East, summed up the situation there:

“*In the East most of the resettled community is fighting for their survival with hardly any structures to support their wellbeing - lack of hospital, school etc. Even if there are buildings, there are few permanent staff members. Further, because of this issue mothers are going abroad to earn for their family but leaving their children. Another issue of concern in the Batticaloa district is the alarming increase in suicide in the absence of diagnosable mental illness. Females are committing suicide by hanging. Hanging as a mode of deliberate self-harm is also increasing. Most of the suicide attempts we have observed, there is either an associated child abuse or gender based violence as one of the primary issue*”.

Such comparisons with the situation with the East are important to understand similarities and differences in the post-war problems and contexts.

Ex-combatants faced many difficulties with reintegration back into society and finding gainful employment. A Sinhalese KI, a keen observer of developments in the North, observed referring to the breakdown in traditional cultural values:

“*Hopefully a ‘new’ culture will emerge from the younger people, if and when the ‘development’ of the physical environment that is promised (and it seems to be taking place) takes root ­ and perhaps if professionals and teachers and even government servants provide the structure for psychological development to take place, ­ especially instilling or promoting a sense of hope. It may not be the same ‘culture’ that was there in the past but one that takes on board ­ and comes to terms with ­ all the trouble the people have endured. Recovery whether from personal illness or community disruption must happen, ­ always does, ­ if perhaps people can see some hope; giving up hope must not I think be allowed to happen*”.

Hope is a key ingredient of resilience and economic recovery [113].

Positive resilience factors that was observed and were mentioned in the FG discussions included functioning families; dedicated and experienced government and non-governmental officers, teachers and community workers; cultural and traditional beliefs, practices and rituals; commitment to education; and creative potential in narratives, drama such as the traditional *koothu*^b^ and other arts. A KI from the Education Department, described the relatively quick resumption of educational activities and reestablishment of basic school structures and functioning in the IDP camps and resettlement process. He pointed out that initially students performed extraordinarily well in national exams despite the hardships they had undergone and limitation for study after displacement and resettlement. However, he pointed out that education standards had deteriorated rapidly thereafter, with the Northern Province ending up at the bottom nationally, though there have been some outstanding individual performances. He attributed this fall to lack of motivation, limited opportunity structures and social overvalued emphasis on exams and tutorial method of study.

Despite the restrictions on public performances and expression of grief, IDP’s spontaneously turned to *Koothu* to express what had happened to them after the recent Vanni war. After resettlement, *Kovalan Koothu* was performed all over the Vanni with large attendances and community participation. In the traditional folk form of *Opari* (lament), recent experiences and losses from the Vanni war was incorporated in to community grief performances [114]. In terms of progressive development there were new ways of thinking, breaking traditional boundaries; female leadership and agency and their acceptance by the community, their empowerment and emancipation from earlier repression; entrepreneurship, ‘*awareness of global trends, emerging trends in adapting different forms of artistic expressions (like cinema and short films)’ , reduction in caste barriers, multi-cultural milieu, rights oriented thinking and behaviour*’. The presence of functioning family units, village and community structures, networks and culture were protective while their absence were risk factors.

There were signs of re-emergence of collective efficacy in some extreme cases. Apart from the aforementioned remembrance observations and earlier community action during the ‘*grease putham*’ scare, in May (2012) the community in Karainagar, particularly the youth, had taken matters into their own hands when an intellectually disabled girl was sexually abused by visiting salesmen who happened to be Muslim. The Muslim community in Jaffna immediately condemned the actions of the salesmen. However these were extreme situations where the community was pushed to respond to challenges to its values, emotional needs and security. Our observations and participant reports indicated that most people felt they had no control over what was happening. Decisions were taken higher up or in Colombo and implemented without consulting them. People had lost trust in the state institutions. To rebuild community resilience and social capital, civic engagement and responsibility in day to day affairs and a general confidence that community action will bear fruit would have to be re-established. However, this may not be possible where the locus of control is dependent on external authorities.

An academic and community activist mused,

As academics and community leaders, we have to look for the available positive features which are still surviving after exposure to a terrible protracted war for more than three decades. We need to empower the communities rather than empowering the politicians or individuals. We still expect the state to provide everything, that means we still depend on politicians. We need to have self-sustainable communities in every part of the country. State mechanism should depend on these communities.

Some common issues raised by the participant included noticeable increase in suicides and attempted suicides as indicative of serious psychosocial problems and unresolved grief and governance as inhibiting community recovery and resilience

### Suicide

**Figure 3:**
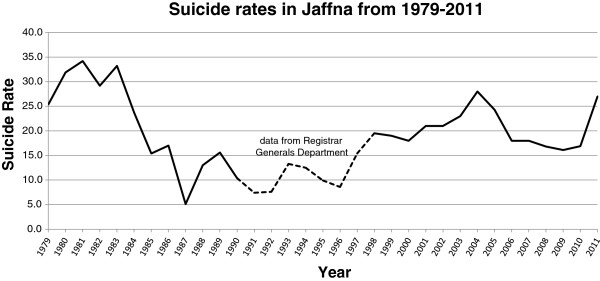
Suicide Rates in Jaffna (Obtained from Jaffna District Courts. 1990–1997 figures from Registrar Generals Department).

There was a general perception and concern about the alarming increases in self-harm and suicides attributed to various psychosocial and economical causes in the post-war context. Although suicide appears to be a personal, individualistic action, it has broader social ramifications. Suicide rates have been used as a parameter to reflect the health status of societies. The sociologist Durkheim [115] showed that suicide rates are remarkably constant for each society, but varies according to socio-economic conditions. He demonstrated that there is a marked fall during war. Suicide rates in Jaffna too have shown the same trend with a marked fall during periods of intense fighting [116] and an increase during cessation of war or peace (see Figure 3). According to Durkheim, war increases social cohesion against a common enemy and this gives meaning to life. However, those who may commit suicide during normal times may die from other causes during war. Neeleman [117] described the phenomena of ‘contextual effect modification’ with the context of war modifying the expected suicide risk by opening up other ways of dying such as enlisting and fighting on frontlines. Thus, the drop in suicide rates could instead be due to war providing an alternative channel for suicidal behavior, but Burvill [118], rejected that explanation based on the figures from Australia. The psychodynamic explanation describes suicide as similar to depression - a form of aggression turned inwards towards the self, whereas war provides an outlet for the aggression to be turned outwards towards a common enemy [119].

Suicide and Attempted Suicide in the Killinochchi District also is increasing, though suicides appears to be a plateauing in 2012 while attempted suicides continues to rise (Tables 2 and 3). The Killinochchi Hospital statistics do not represent overall figures for the whole district which would be substantially higher, but they do show patterns that can be discerned. It is not clear what the sources for the Registrar General Department figures are and how accurate they would be, but are included for comparison and understanding trends.

**Table 2:** **Suicides in Killinochchi** (**Killinochchi District General Hospital** (**DGH**) &**Registrar General Department**)

**Year**	**Number**		**Population**	**Crude Rate**^**1**^**(per 100,000)**	
2003^+^	2	(40)			(28)
2004^+^	26	(24)	141843	18	(17)
2005		(54)			(38)
2006		(29)			(20)
2007-9	Not available				
2010	29		115,417 (Nov., 2010)^*^	25	
2011	42		121,318 (Dec., 2011)^*^	35	
2012 ( first six months)	19		124142^*^	30.6	

^1^Crude rates- Though not strictly reflecting district rates, calculations were done from Killinochchi Hospital data and where available Registrar General Department data to give an idea of trends. The number of suicides for the district would have been higher than the hospital figures, perhaps reflected in the Registrar General Department figures. During the war there would have been problems of transport and other difficulties so that the war and post war contexts would be different. However, the period 2002 to 2006 was the peace process’ time where some of the contextual factors were similar. It is not clear the source of the Registrar General Department figures but it is noteworthy that the numbers for 2004 are almost similar.

^2^ ( ) Registrar General Department data within parenthesis.

^+^ Jeyakumar, J. (2005) Study of Suicide and Attempted Suicide in Killinochchi, Killinochchi Medical Association, Killinochchi.

*Population figures from the United Nations Office for the Coordination of Humanitarian Affairs [75]http://www.hpsl.lk/Catalogues.aspx?catID=74.

N.B. Sri Lanka national suicide rate was 20 in 2006 and 20.6 in 2010. The average Global Suicide rate is 16 per 100,000. Worldwide suicide rates by country can be found at WHO [http://www.who.int/mental_health/prevention/suicide_rates/en/].

**Table 3:** **Attempted Suicides** (**Killinochchi DGH**)

**Year**	**Number**
2000^+^	26
2001^+^	80
2002^+^	68
2003^+^	96
2004^+^	186
2010	180
2011	331
2012 (six months)	177 (354- projected)

^+^ Jeyakumar, J. (2005) Study of Suicide and Attempted Suicide in Killinochchi, Killinochchi Medical Association, Killinochchi.

Thus the current trend of increasing suicides and attempted suicide can be expected to rise further in the post-war context unless energetic and effective remedial measures are taken to ameliorate the socio-economic and emotional deprivation and create opportunity structures and hope for youth. The following case of suicide in a University of Jaffna student that received wide media attention [120] illustrates the complex war and post-war dynamics involved:

*P. was a final year arts faculty student. She had been born in Killinochchi to a family with many siblings. When she was six her father had been arrested by the LTTE and branded a traitor to the nation (thesa throhi). He had been paraded in road junctions with announcements over loud speakers all over the north with a placard hung from his neck with the words, thesa throhi. He was then disappeared by the LTTE but her mother continued to search for him for over five years. They never got to see his body. P. grew up in these circumstances in a stigmatized family. She had been a brilliant student at Killinochchi Mahavidyalayam. She was of a sociable and generous nature helping other students in their studies. She gained admission to Jaffna University in 2007 but the LTTE refused to give her the ‘pass’ to leave their area. She was forcefully conscripted by the LTTE in 2009 and her hair shorn. While serving on the frontlines she had sustained multiple shell blast injuries. Her mother was killed and sister fatally injured by the shelling at Mullivaikal. When she later learned that her mother had been killed, she had developed a deep grief from which she never recovered. She was treated at the welfare centre of the IDP camps for her shell blast injuries. Her previous university admission enabled her early release from detention. Without any close family or social support (her sister married against her wishes and she did not maintain contact but had given her all the family jewelry and land), she initially stayed at the university hotel. Economically she was helped by funds from a distant cousin who was abroad, and other sources. The parents of the cousin were not happy with this help. She had been quite depressed, given to crying, expressing suicidal ideation, wanting ‘to go where her mother had gone’, listening repeatedly to songs with the theme of mother, and gazing at her mother’s picture. She was said to be a loner, not taking part in usual activities, but would share and find solace by talking about her situation to hostel mates. The hostel environment was believed to have been supportive and nurturing. Retrospectively, it can be suspected that she was suffering from prolonged grief, post traumatic symptoms and depression. However, due to increasing numbers being admitted from elsewhere, the university restricted hostel places to first and final year students*. *She felt compelled to move out to a room where she became isolated and would lock herself up in the room on returning from the lectures. During the Easter holidays when most students had gone back to their homes, she had gone to an aunt in Killinochchi and then to Vavuniya. After discretely donating whatever she had to elder’s homes and others and attempting to return the funds in her bank to her relations, she had gone to a nearby well and committed suicide there.*

A mental health professional confessed about this tragedy, “*Suicide is a social issue and social factors are not favourable to support needy people. It is a wonder that more people are not committing suicide in this hopeless and helpless atmosphere. It could be a sign of their resilience to survive in these conditions”.* It was remarked in media reports that earlier too, a university student with Mullivaikal experience (where the majority of the population of the Vanni were trapped by the earlier fighting) had committed suicide. In the feedback discussions, a KI pointed out a University of Jaffna Community Medicine Research study of university medical faculty students from the Vanni in 2010, which found 82% had been directly exposed to the war situation, 67% had barely escaped death, 35% had lost family or friend, 43% had witnessed killing, 27% had been imprisoned, 23 % kidnapped or abducted, and 18% had been tortured or beaten [121]. Such high levels of exposure to traumatic events will need recognition, special care and necessary support.

### Grief

A very common psychosocial problem that emerged from the interviews and FG discussions was unresolved grief. Many had lost a family member, kin or close friend under traumatic conditions. Sometimes the death had been painful with untreated injuries and bleeding. Some had to abandon the body without performing traditional rites while others had to leave them before death for their own survival. Some family members had been separated, detained or disappeared leaving a lasting ambiguity and lack of closure. Survivors often had no acknowledgment whether the person was alive or dead and about their circumstances. Anniversary observations and community observance of traditional practices for the dead have not been permitted under the present authorities. Many had guilt over not carrying out the customary rituals, remembrance observations and other practices to honour the dead, socially establish their passing away and get on with life. Organizations and community groups had helped to arrange for the rituals in covert and increasingly overt ways that signaled resilience. The recent (2012) observations of the deaths at Mullaivaikal, in defiance of the authorities, were an expression of collective grief. A mechanism will have to be evolved for dealing with the unresolved grief, such as cultural rituals, public acknowledgement of the deaths and monuments to commemorate their death.

### Governance

**Figure 4:**
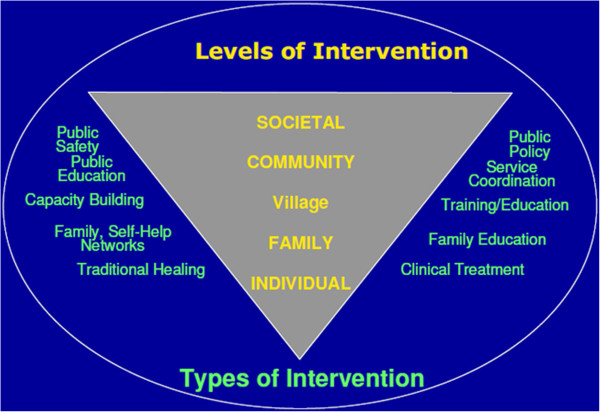
**Levels of Interventions **[2]**.**

Although not within the original research objectives where individual, family and community level changes were to be studied; governance, clearly an issue pertaining to overall public policy and administration (see Figure 4) was brought up consistently in the KI and FG discussions and was clearly noticeable in the day to day functioning of people, institutions and administration. As authentic research and in the interests of finding effective measures for rebuilding community resilience, these expressions could not be left out of the report despite the risks. All the participants and PS workers expressed relief at the end of the violence, deaths, injuries, and displacement that had characterized life for over two decades and clearly did not wish for a return to those conditions; they said the current militarization and administrative control from Colombo was hampering the recovery of community agency. There was a sense that the military was directly or indirectly involved in all matters of their life and final authority for decisions came ultimately from Colombo. The Governor and Presidential Task Force (PTF) were mentioned repeatedly as ultimately deciding on all economic and developmental programmes and projects. There was a general belief that psychosocial programmes and counselling were not allowed. Community work; organizing traditional rituals, remembrances, celebrations, festivals, meetings, community discussions, organizations, groups; beginning socio-economic programmes and livelihood projects needed prior clearance, and were closely scrutinized and monitored. Several workers confided during their interview that they had been questioned about the nature of their work by persons identifying themselves as ‘state intelligence’. People felt powerless over what was happening to them and helpless about initiating any constructive activity. There was uncertainty and hopelessness about the future and lack of a sense of control. A KI, an international WHO expert who had also worked in the Middle East, said in relation to building lasting peace:

"*The present militarization and restrictions placed on the population (in the North and East of Sri Lanka) reminds me of a similar situation the Palestinian Diaspora face in Lebanon, the West Bank and Gaza. There is a constant cycle of fighting (armed rebellion by 'Martyrs') followed by defeat and initial despondency and many of the features of population trauma now witnessed in the North and East of Sri Lanka, particularly in the Vanni. In the Middle East, this state of affairs is then usually followed by anger and resentment and eventually armed uprising. I was told the youth are also now more and more attracted to the more radical Al-Qaeda type groups. As one Doctor in Shatila Refugee Camp (in Beirut, Lebanon) mentioned to me 'when you have nothing, you have nothing to lose'.*

*In contrast the very large Palestinian Diaspora in Jordan (where they have full citizenship, no barriers to employment and very few restrictions placed on them by internal or external security forces) do not follow this cycle of violence*."

Many of the participants had faced difficulties in adapting to the current legal and administrative requirements and structures that had been in place before the conflict, having become accustomed to a different set of rules and functioning when under the (now defeated) LTTE. Some complained that local state authorities were reasserting their control and reestablishing earlier ways of doing things. Many were consequently frustrated and depressed by bureaucratic requirements, delays, what appeared to them as arbitrary decisions, undue influence and exclusion from benefits. They felt the actual needs of the populations were not given priority but were being addressed through regular channels. In the Vanni which had been reduced to something resembling ground zero (and with large shortages of human resources), plans and appointments were handled through routine methods. In our participation observation, the appointment of one Medical Officer of Mental Health (MOMH) for the whole district took persistent requests, and much effort and time though he was willing and had started to work there on a volunteer basis without salary. The Mental Health Consultative Forum, Northern Province chaired by the Provincial Director of Health Service and having representatives from all the stakeholders in the government and NGO sectors, of which the authors were a part, met several times at the beginning of IDP resettlement process came up with a detail Mental Health Plan that was ignored and the committee made defunct. ‘Punishment transfers’^c^ were mentioned as a common method of administrative control. There was a general feeling among those interviewed for the study that officers who went out of their way to help the public or looked at the problems through a humanitarian perspective rather than following official dictates were sent on punishment transfers. Many of the participants felt that the study, feedback discussions and documentation gave them a voice, an opportunity to express their observations and recommendations, emboldened albeit anonymously, that hereto they had felt afraid to speak about or only talked about among themselves.

Community observation of caste differences and patriarchal norms was reported to be resurfacing. Women were being compelled by circumstances, like female headed households with responsibilities for the family, to negotiate with the authorities. In the process they were facing hardships, discrimination, harassment and abuse. Authoritarian personality^d^ functioning, hierarchical control and obedience, competition for favours, patronage and informing on local matters to higher authorities were observed in administrative functioning. Many administrators were beholden to Colombo, the governor and the military and tried to avoid displeasing them. A KI described the power dynamics as follows:

“*The Governor was the ex-military commander of Jaffna. During that period, all sides used extrajudicial killings and disappearances to control the population. People lived in terror in those times. People are still having that fearful image and are afraid of adverse consequences other than the routine civilian way of punishments. Though there are much fewer incidents, it appears that the same military style of administration to control the civilian structures continues*. *Moreover, the same military that was responsible for the human rights abuse during the war are now placed in total control over the local population. This does not enable a trustful relationship and hopeful environment to develop*”.

On triangulation, confirmation for the ecology of terror during that time comes from University Teachers for Human Rights (Jaffna) [95,96,107] and wikileak documents [98]

In the principal author’s effort to obtain ethical clearance from the University of Jaffna for this and the previous study of the Vanni IDP’s, the ethics committee has been hesitant to grant clearance. A KI, a young researcher from the University said, “*Most of them are not very enthusiastic and a few were even discouraging! I don't know what's behind all these. But, a few of them have encouraged me and told me to submit the proposal in a way, that can assure no blaming on the Government regarding war issues and few even told to me avoid the word 'war'*”. Another academic added, “*Several small scale research projects on mental health issues by students also had difficulties in getting ethical clearance for the same reason*”.

The psychiatric department at Jaffna University has been closely monitoring suicide rates from the early 1980’s (Figure 3) to understand trends and take preventive measures. The courts had always cooperated in making available their coroner’s verdict records, but from this year (2012), some courts have started to insist on first obtaining permission from the Judicial Services Commission in Colombo, a cumbersome procedure. According to professional KI’s from Jaffna, “A *request to meet the judges who are working in Jaffna and discuss pressing local issues was rejected by the Judicial Services Commission*”. In the middle 1980’s when civilians had been killed by bombing raids, enterprising judges ordered arrest warrants on the perpetuators, the Sri Lankan Air Force pilots. The police never carried out these arrests and some judges were transferred. When the Indian Peace Keeping Force took over Jaffna in 1987–88, they tried to take control of the civil administration, but the local administrators resisted through subtle non-violent ways and the Indians finally gave up. A KI, a health specialist, said that the “*irony of current developments after the war was that those who were at the forefront of administering under the LTTE, had now changed over to working for the government using the same authoritarian style*”. Though an authoritarian style of functioning was characteristic of traditional Tamil culture, under the present circumstances of recovery after almost three decades of war and devastation of social capital, it becomes an obstacle to rebuilding resilience. A KI, a doctor working in the health sector observed, “*most top administrators in important institutions are political appointees who carry out their agenda, creating a host of problems within the community*."

The power differentials, lack of control and militarization which are critical issues in the North as several recent well researched reports of the ground situation [74,122,123] have pointed out, are beyond the scope of this study. This study focused rather on internal and local community characteristics, processes and functioning to promote resilience despite the aforementioned constraint at the higher policy level (see Figure 4 for levels of interventions). However, an observation by Darini Rajasingham-Senanayake [124] sums up the importance of addressing these aspects:

*“Militarization has increasingly deformed democratic institutions and processes, while foreclosing possibilities for reconciliation and closure and thus leaving space for endemic conflict. In the context it is worth noting that a primary reason that Sri Lanka had and still has the best social indicators (health, literacy, education) in the region was the early post-colonial investment in social infrastructure and the welfare state, rather than in the defense sector by leaders of the independence movement in the island. Now that the war is over, Lanka needs to once again lead the way in South Asia: demilitarize and reinvest once again in human resources and the social sectors, particularly education, and ensure power-sharing among the island’s diverse communities”*.

There is an urgent need to change from the war and post-war military mode of thinking and administration to a post-conflict civilian authority so that an enabling environment, the open space for the reemergence of community resilience can be engendered.

## Conclusions

Many of the psychosocial and mental health problems that were observed and noted in the case studies, KI interviews and FG’s had been prevalent even before the war and could be seen in normal times, but the post-war context was exacerbating and in some cases, causing them. The common theme running through all the narratives, though not always mentioned verbally but lurking in the background, were the war time experiences, losses and post-war context which formed a common denominator to the psychosocial issues facing the general population. The memories of what had happened and the emotions they had felt were still affecting their behavior as much as the ongoing post war context and stressors. These issues have to be addressed if the community is to recover and benefit from rehabilitation and development programmes [77]. Most of the problems and observations brought out in the case studies, FG and KI interviews, confirmed by organizational and media reports, were of a negative nature. There was a sense of loss of communality and agency. People were resigned, passive and silent. The state and military were in control, determining all matters. People felt beholden and dependent. There was a general consensus that if these psychosocial problems could be addressed adequately communities would recover. Unfortunately, a conclusion of this study is that the ecological context is not conducive in the post war setting for community recovery, development and growth. Despite this adverse environment some individuals, families and communities were surviving and coping creatively. The study has found some resilience and adaptation. Our aim should be to encourage these positive developments and create an ecology that would engender resilience. Towards this end, the following recommendations are put forward based on experiences reported in the literature from similar post-war situation around the world, best practice principles from international organizations like the WHO, experiences gained during the war and after the tsunami [91] as well as what came out during the study and feedback discussions:

## Recommendations

### Community based programmes

In view of the widespread nature of the mental health and psychosocial problems in the population as shown by several local surveys [82,83,125], findings from this study and the low resource, post war context; the best way to address the problems would be a community based, public mental health approach. Apart from the equivocal evidence on the efficacy of individualized approaches based on medications and CBT for psychiatric conditions such as PTSD [27], particularly in non-western settings, and the lack of appropriately trained professionals in a resource poor setting; the supraindividual trauma at the family and community levels in a collectivistic society would be best addressed through a community based approach that would reach the largest population. The North, East and border areas have undergone a disaster of enormous proportion. As after the Tsunami, a large relief, recovery, rehabilitation and development effort to address the needs of this population has to be launched. A good guide that could be implemented is what has been recommended in similar situations by the WHO [36] and other international humanitarian organizations like the minimum standards of the Sphere Project [37] and the IASC guidelines [38] as was done after the tsunami in the North [103,126]. The IASC recommends that after addressing basic needs like food, shelter, security, basic health care; a Mental Health and Psychosocial Support or ‘MHPSS response in participatory, safe and socially appropriate ways that protect local people’s dignity, strengthen local social supports and mobilize community networks’ (see Figure 4) should be implemented.

When the draft findings and recommendations were fed-back to the participants and key-informants, one responded emphatically:

*This is an important step that we all should have in our mind. There is a need to respect other people’s dignity in all the situations, trivial or crisis. The recent past has imprinted the inhumane and authoritarian approach towards a fellow human. We need to consciously improve our humane qualities and this will help us improve our self-image, relationships, interactions, social support and network and of course our resilience. Similarly, trying to provide a chance for others to express their views, consulting all the stakeholders during decision making and developing a culture of dialogue, negotiation and forgiveness in all our life situations whether at home, at our work place or at our community, will help us to heal from our past and move forward. These things may look like unimportant tiny little things but they serve their purposes and help the community towards constructive development*.

Other organizations working in similar confict and post-conflict situations around the world have used the MHPSS approaches [9,31,127,128].

The IASC [129] states that ‘*in most emergencies, there are significant disruptions of family and community networks due to loss, displacement, family separation, community fears and distrust. ..Useful responses include family tracing and reunification, assisted mourning and communal healing ceremonies, mass communication on constructive coping methods, supportive parenting programmes, formal and non-formal educational activities, livelihood activities and the activation of social networks, such as through women’s groups and youth clubs’*. During the war and after the tsunami, we tried to do this through increasing local awareness and transferring knowledge and skills of how to deal with common mental health and psychosocial issues to local resources [87]. This was done through training of community level workers and human resources in villages. At the same time, strengthening and expanding existing resources and capacities; capacity building of primary health care workers to deal with common mental health issues; and engendering local participation, leadership, decision making, planning and implementation to rekindle collective hope, trust and efficacy are urgently needed to rebuild community agency and resilience. An innovative community based programme was designed by the WHO after the tsunami by training large numbers of Community Support Officers (CSO’s) to look after the mental health and psychosocial needs of families and implemented effectively all over the affected areas in Sri Lanka [130-132]. They were mobilized to work in the IDP camps in Vavuniya in 2009 and are being deployed in the resettlement process but in limited numbers. The numbers and training needs to be increased.

During the feedback on the recommendations, a KI who had been involved in the training of community resources commented that once they had the knowledge and skills:

*This could be used in almost all the day to day life settings. Teachers can incorporate some aspects of the mental health and psychosocial issues during their teaching sessions. Lecturers can talk about romantic affairs and how to have safe relationships and how to deal with frustrations without choosing a life threatening self-harming method. A priest can talk about improving the family dynamics and an artist or the media can promote the positive aspects of life*.

The study found that the war and post-war contexts were impacting negatively on the family and extended family due to deaths, disappearances, separations, displacement and poverty. As the functioning family is the basic building block and foundation of Tamil communities, it would be essential for the community workers to promote the restoration of functioning family units. They could work with families to help them trace missing members, partake in cultural grieving ceremonies for the dead, improve relationships and correct misunderstandings among members, reestablish hierarchical responsibilities, create income generating opportunities for the family and generally encourage unity and positive dynamics. Problems of domestic violence, child abuse, alcoholism in the male, unwanted pregnancies, extra marital relationships, suicide and self-harm, the elderly, and widows could be addressed within the functioning family structure as well as at the community level.

The evidence for collective trauma found in the study can be approached through community based programmes to promote the positive resilience and adaptive coping strategies as outlined below. A sense of agency, control, determining their own future and a belief in their collective efficacy had to be restored to the families and communities (Figure 1).

It is only by creating a sense of community, collective efficacy and confidence that social capital can be increased, leading to a gain cycle [48] where trust, motivation and hope can be re-established. Linking social capital where communities have access to power, decision making and resources are vital for building resilience. At the same time, there is a need to also address the negative aspects like lack of trust and uncertainty. Efforts will need to be directed at rebuilding social capital through community networks, relationships, responsibilities, roles and processes.

To address the finding from the case studies of extensive poverty and lack of avenues for socio-economic advancement, the community workers have to work towards creating opportunity structures for education, vocational and skill training, and capacity building particularly for youth and income generating programmes through networking with other governmental and non-governmental (NGO) agencies. It is by establishing some economic stability, livelihood and access to resources that families and communities will regain their dignity, faith and hope. Improvement in mental health and psychosocial wellbeing would motivate the population and enable better participation in rehabilitation and development programmes, that was found to be clearly lacking in this study.

### Cultural rituals and ceremonies

It was significant that the study found the communities, though under considerable restrictions and monitoring by the military, had resorted to traditional practices like *opari and koothu* to express their grief and find solace. It can be expected that communities will regain their natural resilience when performing customary rituals, observe ceremonies like remembrance days and partake in community gatherings and festivals. Encouraging and teaching cultural relaxation methods at the community level is one way we have done this in the past [133]. This would give some relief from the grief and guilt, create faith, meaning and social support and networks. Community monuments that would help focus and express emotions after mass trauma have been called traumascapes [134]. For example, a civil monument at Mullivaikal to all who died there (military, militant, civilian) by a sensitive sculptor and national ceremonies to be observed there annually would go a long way towards reconciliation.

**Figure 5:**
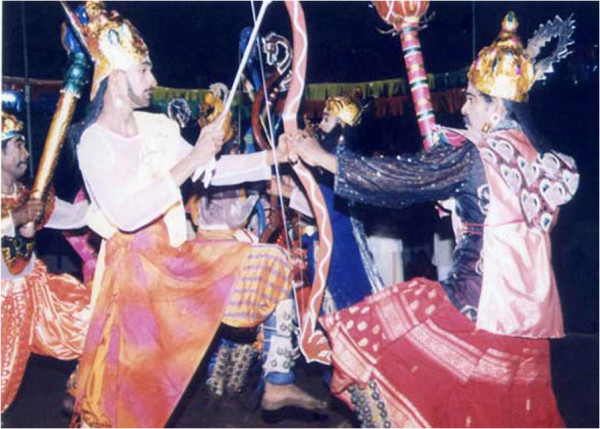
***Koothu.***

As we found during the war and after the tsunami, creative arts are valuable conduits for the expression of emotions, finding meaning and developing meaningful community narratives [7]. *Koothu* (see Figure 5) [135], other dramatic forms, *laments,* poetry, writings and drawing should be encouraged and promoted.

### School based programmes

As observed by a KI, one of the first positive structure and activity during the recovery phase in IDP camps and resettlement was education related activities. The importance given to education in Tamil culture should be promoted and used to re-establish old routines, structures, and hope in the future. Teacher counsellors can function as community workers to organize activities in the classroom and schools such as play, group activities, creative endeavors, and inter-communal relationships. Several school based programmes were implemented during the war and after the tsunami where teacher counselors and befrienders were trained from all over the North and East [86,136], who could now be used for the current situation.

### Allow and assist families and communities to settle in their own familiar homes and villages

The case studies and observations showed that families were eager to return to their familiar surroundings, home, neighborhood and villages. Some were shocked when confronted with destroyed homes and bare land. Resettling in their ancestral home, village (*uur*) or region (*thesam)* will have healing effect [108] and build resilience [77]. The traditional Tamil identity based on *veedu* and *uur*[109] may need to be re-established in the post-war context.

Overall, long-term psychosocial programmes are not only important for restoring well-being and functioning, but also to rebuild social capital and community resilience which would enable and empower affected communities to help themselves.

## Endnotes

^a^*Grease putham* refers to folklore about male(s) smeared with grease or oil (to make it slippery to hold onto when apprehended) attacking females at night.

^b^K*oothu is a form of traditional, folk drama that is performed with music and dance with religious, epic and historical themes where well known characters express common themes. In the Vanni, a form called Kovalan koothu is popular and performed by adepts who have usually been trained from childhood by elder practioners.*

^c^When state officers do not perform, carry out expected duties or do not obey directives they are transferred to less favourable posts, usually in the periphery.

^d^Authoritarian Personality is characterized by unquestioning obedience to those above and strict control of those below in a hierarchical structure with structured lines of authority where order, power and status are valued.

## Competing interest

The authors declare that they have no competing financial interests but are Tamil medical officers working in northern Sri Lanka with an abiding interest in the welfare of the region and people.

## Authors’ contributions

DS functioned as the principal author involved in designing the study, collecting and analyzing the data and writing the manuscript. SS functioned as the co-author, contributing towards conceptualization, context analysis, cleaning the data and reviewing the manuscript. Both authors have read and agreed upon on the final manuscript.

## Authors’ information

DS, was Seniour Professor of Psychiatry at the Faculty of Medicine, University of Jaffna and Consultant Psychiatrist working in Northern Sri Lanka for over two decades. He has also worked in Cambodia for two years in a community mental health programme with the Transcultural Psychosocial Organization. He is a Fellow of the Royal College of Psychiatrists, UK; the Royal Australian and New Zealand College of Psychiatrists and Sri Lanka College of Psychiatrists. Currently on extended sabbatical in Australia, he is a consultant psychiatrist at Glenside Hospital, Supporting Survivors of Torture and Trauma (STTARS) and Clinical Associate Professor at the University of Adelaide. He hopes to return to Jaffna this year.

SS is a consultant psychiatrist working in Jaffna with the Doctor of Medicine (MD) in psychiatry from the Post Graduate Institute of Medicine, University of Colombo. He has worked in the North for over a decade clinically and in community programmes. He is a graduate of the Jaffna University medical faculty.
